# Exploratory and confirmatory factor analysis of the 12-item Arabic World Health Organization Disability Assessment Schedule (WHODAS 2.0) as a screening tool for Syrian refugees

**DOI:** 10.1192/bjo.2021.1017

**Published:** 2021-10-11

**Authors:** Aemal Akhtar, Pim Cuijpers, Naser Morina, Marit Sijbrandij, Richard Bryant

**Affiliations:** Department of Clinical, Neuro and Developmental Psychology, Vrije Universiteit, the Netherlands; and School of Psychology, University of New South Wales, Australia; Department of Clinical, Neuro and Developmental Psychology, Vrije Universiteit, the Netherlands; Department of Consultation-Liaison Psychiatry and Psychosomatic Medicine, University Hospital Zurich, University of Zurich, Switzerland; Department of Clinical, Neuro and Developmental Psychology, Vrije Universiteit, the Netherlands; School of Psychology, University of New South Wales, Australia

**Keywords:** Psychosocial interventions, low- and middle-income countries, factor analysis, disability assessment, validation study

## Abstract

**Background:**

The World Health Organization Disability Assessment Schedule 2.0 (WHODAS 2.0) is a generic measure of functional impairment and disability but to date no studies have reported its applicability in a population of Syrian refugees.

**Aims:**

The aim of this study was to explore the psychometric properties and factor structure of the Arabic version of the WHODAS 2.0 among a population of Syrian refugees in a Jordanian refugee camp setting. The tool was used as part of a screening procedure for a randomised controlled trial assessing the effectiveness of a low-intensity psychological intervention.

**Method:**

A representative sample of Syrian refugees (*n* = 650) were screened to assess levels of functional impairment and psychological distress. The screening results were used to explore the internal consistency and dimensionality of the WHODAS 2.0. We assessed level of convergence with the validated Kessler 10-item Psychological Distress Scale (K10), which assesses psychological distress. Exploratory factor analysis (EFA) and confirmatory factor analysis (CFA) were conducted to explore the construct validity and factor structure of the WHODAS 2.0.

**Results:**

The mean baseline WHODAS 2.0 score was 20.5 (s.d. = 7.6). The internal consistency was acceptable (Cronbach's alpha 0.74), with all 12-items appearing to be related to the same construct. The WHODAS 2.0 was positively correlated with the K10 (*r* = 0.57, *P <* 0.001). The results of the EFA identified a three-factor solution accounting for 51% of variation, corresponding with factors related to self-activities, external activities and self-care. CFA results indicated good fit of the three-factor solution.

**Conclusions:**

The results indicated that the WHODAS 2.0 has a three-factor solution and is an acceptable screening tool for use among Syrian refugees.

## Background

Refugees face a myriad of pre- and post-migration stressors that have an impact on their mental health and well-being.^1^ Levels of disability and functioning in refugee populations are moderated by both environmental and biopsychosocial factors. Environmental factors, such as limited access to basic needs and harsh living conditions, can lead to reduced abilities to function.^2^ Psychosocial distress and poor physical health directly have an impact on disability and functioning.^3^ There is a well-known link between emotional distress and related impairments in functioning, meaning those with higher levels of distress are likely to experience more marked impairment and disability.^4^

The International Classification of Functioning, Disability, and Health (ICF) framework defines disability as an impairment in individual functions, activities and participation, leading to a decrement in the individual's ability to function.^5^ Disability can be considered a broad construct spanning several domains of daily life, making it challenging to measure. With the increased focus on the mental health of conflict-affected populations in emergency settings, programmatic initiatives must be able to effectively screen for disability to better inform clinical practice and public health policy. The World Health Organization (WHO) Disability Assessment Schedule (WHODAS) was developed to effectively measure disability across the six domains as outlined in the ICF.^6^

The WHODAS 2.0 is a generic biopsychosocial measure that aims to capture levels of functioning and disability, and aspects of health-related quality of life, across the six domains of functioning: (a) cognition, (b) mobility, (c) self-care, (d) getting along, (e) life activities (household and work), and (f) participation.^7^ There are three versions of the WHODAS 2.0; 12-item, 12 + 24-item and 36-item versions, and it has been widely translated into over 30 languages. Stringent tests performed on the WHODAS have shown it can be used across cultures, genders and age groups, and across different diseases and health conditions, including both mental health and physical disabilities.^8^

The psychometric properties of the WHODAS 2.0 36-item version have been widely documented through the initial development work of the measure,^6^ as well as subsequent research, yet fewer validation studies have explored the psychometric properties and factor structure of the 12-item version.^8^ During the initial development of the measure, the 12-item version was found to explain 81% of the variance of the 36-item scale. The 12-item version is often recommended for contexts that are characterised by time constraints and in turn could be considered an appropriate measure for screening purposes.^6,7^ Because of its brevity and ease of use, the WHODAS 2.0 has been widely used as a screening tool in large-scale studies.^9^ The psychometric properties of the 12-item version have since been explored in both general populations and a variety of clinical settings.^9^ Exploratory factor analysis (EFA) of the 12-item measure have found varied results as to the number of factors with results varying between one and five structures. Predominantly though, results of EFA feature one- and three-factor structures across diverse populations.^10–19^ Confirmatory factor structures of the 12-item version have been more seldom explored and often constrained to either a one-factor structure with the individual items falling onto one global disability factor or the original second-order structure of the 36-item version with six domains of disability loading onto one disability factor.^15,18–32^

With the increase in global mental health research in humanitarian contexts, it is imperative that commonly used psychological measures are validated to provide evidence for future use.^33^ Although the WHODAS was developed to allow for a standardised way to effectively measure disability cross-culturally,^22,34^ no studies have reported its applicability in a population of Syrian refugees so far. Additionally, the psychometric properties of the WHODAS have not been explored in populations detained in refugee camps, where specific stressors may have an impact on functioning and how it is measured, such as poor living conditions, limited mobility, unmet basic needs and restricted social access.^35,36^

## Aims

The aim of this study was to examine the psychometric properties of the Arabic 12-item version of the WHODAS 2.0. More specifically, we aimed to conduct an EFA and confirmatory factor analysis (CFA), as well as exploring the reliability and internal consistency of the WHODAS 2.0 as a screening tool in large sample of Syrian refugees residing in a camp in Jordan.

## Method

### Setting and participants

There are approximately 650 000 Syrian refugees registered in Jordan, of which 125 000 (20%) reside in one of three official refugee camps.^37^ Azraq camp is the second largest in Jordan with a population of 36 657 as of June 2020.^38^ There are currently 8660 shelters in use across four residential villages, two of which were used to recruit participants for this study. Participants were recruited between August and December 2019, as part of a screening procedure for a larger randomised controlled trial (RCT).^39^ Potential participants were identified through door-to-door screening of consecutive caravans. One adult from each caravan was subsequently invited to participate in the screening procedure for the trial if they met the following criteria: (a) Syrian refugee, (b) ≥18 years old, and (c) had a child or dependent living in the household aged 10–16 years.

Screening was conducted by assessors who received 4 days training in research ethics, the assessment battery, data collection and general interviewing techniques. Assessments were conducted on a digital tablet to ensure that data could be reliably collected and uploaded. Assessors administered the questionnaires in an interviewer format.

The study has been approved locally by the Institutional Review Board at the King Hussein Cancer Centre in Amman, Jordan and the University of New South Wales Human Research Ethics Committee. Informed consent was solicited prior to participation in the study; participants completed a written consent form and those who were unable to do this provided witnessed oral consent, in line with recommendations from the WHO. The authors assert that all procedures contributing to this work comply with the ethical standards of the relevant national and institutional committees on human experimentation and with the Helsinki Declaration of 1975, as revised in 2000.

### Instruments

#### WHODAS 2.0 – 12-item version

The WHODAS 2.0 is a general measure of disability, encompassing six domains (cognition, mobility, self-care, getting along, life activities and participation), assessing difficulties people have experienced during the past 30 days. The 12-item interviewer-administered version is an abridged version of the full measure and has two items from each of the six domains. Items are scored on a 5-point Likert scale (0,  none; 4, extreme or cannot do), with total scores calculated by the sum of the 12 items, resulting in a range of 0–48. Higher scores on the WHODAS 2.0 correspond to greater levels of disability. A cut-off score of 17 was adopted as an indicator of moderate impairment as this represents the 90th percentile of impairment based on WHO normative data.^7^ We adapted the WHODAS 2.0 Arabic version, which was last translated in 1999, to the updated 2010 version of the WHODAS 2.0 in accordance with gold-standard translation practices.^40^ Items were translated and back translated by accredited translators, with discrepancies rectified jointly by the research team and an independent bilingual individual with previous experience working with health-related questionnaires. The feasibility and comprehensibility of the adapted version was piloted among Syrian refugees and found to be acceptable (not reported). The measure was completed by participants with the assistance of Arabic-speaking assessors during an in-person screening.

#### Kessler Psychological Distress Scale (K10)

The K10 is a general measure of psychological distress.^41,42^ The ten items measure symptoms of anxiety and depression experienced in the preceding 30 days. Responses are scored on a scale of 1 (none of the time) to 5 (all of the time) with total scores calculated as the sum of all items with a range of 10–50. Higher scores indicate greater levels of psychological distress. The K10 has been validated in Arabic-speaking populations.^43^

### Statistical analysis

Reliability of the WHODAS 2.0 was assessed in the current sample by analysing the internal consistency with Cronbach's alpha coefficient. Following Nunnally's recommendations of reliability, a cut-off score of 0.7 was considered acceptable for research purposes.^44^ In addition, to assess whether the individual items were related to the same construct, the item-deleted Cronbach's alpha coefficients were calculated. Internal consistency was further explored through item-total correlations, correlations between individual items of the scale and the total score.

Convergent validity was assessed by considering WHODAS 2.0 responses in relation to the K10, a general measure of psychological distress; this approach was conducted because of demonstrated associations between common mental disorders and impaired functioning. To this end, convergent validity between the WHODAS 2.0 and the K10 was assessed.

An EFA was conducted to determine the construct validity of the questionnaire, and to assess the number of latent factors. Prior to conducting an EFA, the Kaiser-Meyer-Olkin test (KMO) and Bartlett's test of sphericity were computed to determine factorability and whether an EFA would be appropriate. To determine the number of latent factors of the measure, the scree plot was observed and the number of factors scoring above an Eigenvalue of 1 was used for the EFA. The varimax rotation was used to extract the factors. Items were considered loaded onto a factor using a conservative value of 0.4 for the correlations between items and components.^45^

The results of the EFA were used to inform the model structure of the CFA. As varimax rotation was used for the EFA, correlations between the individual factors were also modelled into the CFA. The final factor structure and results were reported as standardised correlation coefficients. The model fit for the observed data are reported with the root mean square error of approximation (RMSEA), the comparative fit index (CFI), and the Tucker–Lewis Index (TLI). Acceptable model fit was defined as RMSEA <0.08, CFI >0.90 and TLI >0.90; good model fit was defined as RMSEA <0.05, CFI >0.95 and TLI >0.95.^46^ All analyses were performed using SPSS 26.0 and SPSS Amos 26.0.^47^

## Results

There were 650 participants screened as part of the larger RCT. Of those who were screened, 446 (69%) were female, 596 (92%) were married, and the average age was 40.4 years (s.d. = 7.1). The majority of participants had not previously received any formal education (*n* = 146, 22%) or had been enrolled in a basic education certificate (*n* = 381, 59%) which less than 50% (*n* = 179) completed. Very few respondents reported having previously attended post-secondary education (*n* = 16, 2%). The average WHODAS 2.0 score was 20.5 (s.d. = 7.6), with a minimum and maximum score reported of 0 and 44, respectively. There were no missing data for any of the WHODAS 2.0 items.

The overall internal consistency of the measure was acceptable, with a Cronbach's α of 0.74 (Table 1). The ‘item-deleted’ analysis indicated that all items were interdependent and related to one another, with Cronbach's alpha scores ranging from 0.71 to 0.75. When exploring construct validity, the scores of the individual items were significant and positively correlated with the overall score of the WHODAS 2.0 and K10 scores. Correlations for items 10 (0.39) and 11 (0.36) were relatively weak (Pearson's *R* < 0.40) with the other ten items having moderate to strong correlations ranging from 0.40 to 0.62. Correlations were positive when exploring the convergence between the WHODAS 2.0 and K10, with correlation coefficients ranging from 0.18 to 0.35, and the overall scores having a correlation coefficient of 0.57 (*P* < 0.001).

**Table 1:** Internal consistency and convergent validity

Item	Cronbach's α	Item-total correlations – Pearson's *r*	K10^a^
Standing for long periods such as 30 min?	0.710	0.620	0.315
Taking care of your household responsibilities?	0.722	0.529	0.326
Learning a new task, for example, learning how to get to a new place?	0.722	0.547	0.316
Joining in community activities (for example, festivities, religious or other activities) in the same way as anyone else can?	0.722	0.547	0.329
Been emotionally affected by your health problems?	0.720	0.554	0.353
Concentrating on doing something for 10 min?	0.727	0.524	0.327
Walking a long distance such as a kilometre [or equivalent]?	0.719	0.583	0.304
Washing your whole body?	0.730	0.451	0.274
Getting dressed?	0.734	0.403	0.225
Dealing with people you do not know?	0.742	0.387	0.180
Maintaining a friendship?	0.745	0.356	0.263
Your day-to-day work?	0.712	0.606	0.296
Total	0.743	−	0.572

Kessler 10-item Psychological Distress Scale.

a. All values significant at α = 0.05.

We next explored the factor structure of the WHODAS 2.0. The Bartlett's test of sphericity was significant (*P* < 0.001) coupled with a KMO score of 0.782 indicating factorability and appropriateness to proceed with an EFA. Three factors were extracted while maintaining an Eigenvalue >1, which explained 51% of overall variance of the measure. Factor loadings of the individual items were strong, ranging from 0.46 to 0.88 (Table 2). The first factor had six loadings (items 1, 2, 5, 6, 7, 12), all of which could be classified under the umbrella of ‘internal activities’ and explained 27% of variance. A second factor had four loadings (items 3, 4, 10, 11) explaining 13% of the variance with items relating to ‘external activities’. The last factor had the remaining two items relating to ‘self-care’ (items 8, 9) and accounted for an additional 11% of variance. The resulting component matrix and organization of individual items in the corresponding factor variables is logical from a content point of view.

**Table 2:** Rotated component matrix^a^

Item	Factors		
	Factor 1	Factor 2	Factor 3
Standing for long periods such as 30 min?	0.734		
Taking care of your household responsibilities?	0.530		
Learning a new task, for example, learning how to get to a new place?		0.637	
Joining in community activities (for example, festivities, religious or other activities) in the same way as anyone else can?		0.573	
Been emotionally affected by your health problems?	0.692		
Concentrating on doing something for 10 min?	0.463		
Walking a long distance such as a kilometre [or equivalent]?	0.696		
Washing your whole body?			0.866
Getting dressed?			0.881
Dealing with people you do not know?		0.675	
Maintaining a friendship?		0.649	
Your day-to-day work?	0.691		

a. Extraction method: principal component analysis. Rotation method: Varimax with Kaiser Normalization – rotation converged in four iterations.

**Fig. 1:**
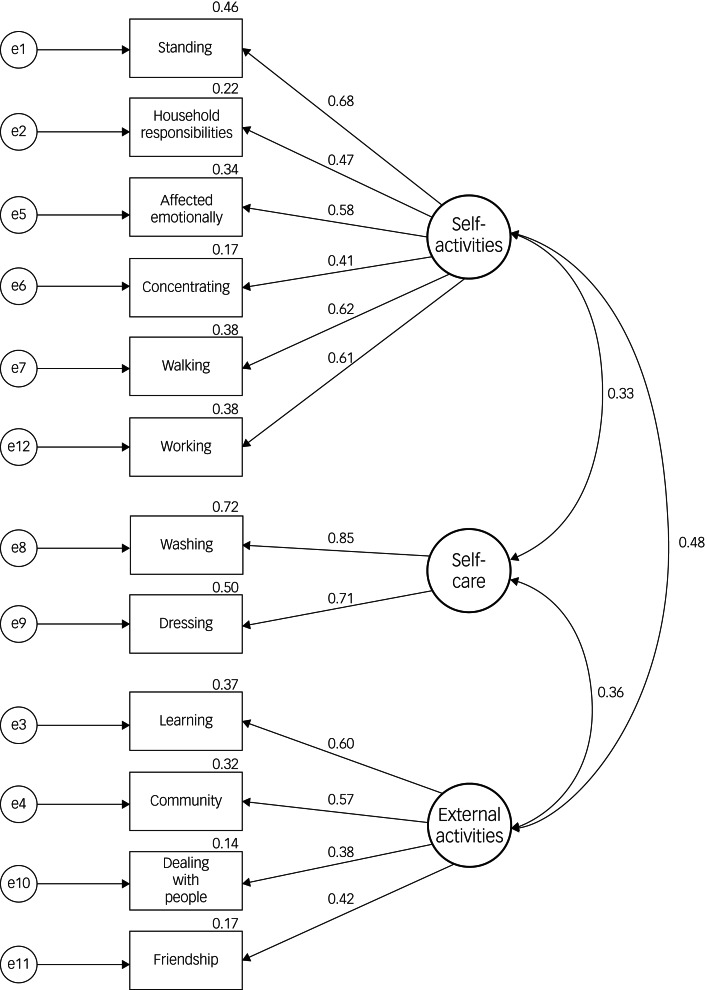
Factor structure of World Health Organization Disability Assessment Schedule 2.0 (WHODAS 2.0) – 12-item Arabic version.

The three-factor structure of the WHODAS 2.0 according to the EFA was used to inform the subsequent CFA. As an oblique rotation was used for the EFA, between-factor correlations were built into the CFA model. The results of the CFA are presented in Fig. 1. To explore model fit, residual covariances were utilised to observe if any loading variables had significant correlations within a factor. Based on low values of modification indices, none were included. The final CFA model fit statistics were good with an RMSEA of 0.043 indicating a close-fit, a CFI of 0.954 and an acceptable TLI of 0.941.

## Discussion

### Main findings and interpretation

These results are the first to explore the validity of using the WHODAS 2.0 as a screening tool to identify impairment in functioning in both a Syrian refugee context and a refugee camp. Using a representative sample, we found internal consistency to be acceptable for individual items and the total WHODAS 2.0 score, and that convergent validity was attained. The EFA presented evidence for a three-factor model, and a subsequent CFA confirmed good fit. The final three-factor model had variables relating to internal activities, external activities and self-care.

Internal reliability assessments suggested all 12-items of the WHODAS 2.0 measured the same construct and that no items in the abridged version should be excluded. It is important to note that the 12-item version was not developed to have separate constructs, rather it was proposed for use as a screener or in contexts marred by limited resources. The Cronbach's alpha score of 0.74 supports the use of the WHODAS 2.0 as a screening measure for disability among Syrian refugees and in a camp-based setting.

When examining correlations in relation to the K10, the WHODAS 2.0 total score had a moderate, yet significant correlation coefficient of 0.57, demonstrating convergence. Two items of the WHODAS 2.0 were found to be weakly correlated whereas the remaining ten were either moderate or strongly correlated. Functioning and psychological distress are distinct constructs, and although we would expect an overlap and in turn correlation between the two measures, convergent validity would have been better explored in relation to other measures of functioning. The moderate convergence might be explained by contextual factors unique to a camp environment. In a refugee camp there are a number of day-to-day factors, such as limitations to mobility and opportunities to socialise, that may directly have an impact on functioning separately from distress. For example, the ability to practice self-care is somewhat limited in camp environments where the focus tends to be primarily on attaining basic needs, rather than engaging in enjoyable activities to maintain well-being. These restrictions that are unique to camp-based settings may explain the moderate convergency between the constructs of functioning and distress.

The results of the EFA indicated strong support for a three-factor structure logically comprising ‘internal activities’, ‘external activities’ and ‘self-care’. In addition, using a conservative correlation cut-off score of 0.4 to determine factor loading, the items were categorised into a single latent factor without overlap. Three-factor structures of the measure have been previously reported in people with anxiety and stress disorders,^12^ motor disabilities,^14^ brain injury^17^ and in older adults in the general population^13^ with a number of similarities in coupled items and factor loading.

The factor structure informed by the results of the EFA, displayed good model fit in the subsequent CFA. The current study only found two indicators measuring the ‘self-care’ factor. Although it is commonly cited that a minimum of three indicators are needed per factor, there are specific circumstances in which two indicators are sufficient.^48^ Given the orthogonal nature of the CFA and that the covariances between the three factors are non-zero, the two-indicator loading of the self-care factor is acceptable. Additionally, given the conceptual logic of the final factors and their respective loading variables, the strong loading for each of the ‘self-care’ indicators (0.71–0.85), and the overall good model fit, having only two indicators for the self-care factor is not considered problematic.

An important consideration of EFA is that the factor structure and number of factors of a measure can vary between different populations, depending on variability in sample selection.^49^ This has been specifically observed for the WHODAS 2.0 12-item version.^13^ Numerous studies utilising the WHODAS 2.0 12-item version, have assumed identical factor structures to that of the 36-item version when conducting CFA. Varying reports of factor structures observed in published EFAs suggests assuming a similar factor structure to that of the 36-item may be problematic. Additionally, constraining the model to two-loading indicators per variable could result in violations of the CFA statistical assumptions.

### Limitations

As this study was nested within a larger RCT, the ability to test for other often reported psychometric properties were limited. As a number of participants who would have screened negative for participation into the trial were not followed up we were unable to observe the test–retest reliability or the sensitivity to change of the measure. In addition, we were not able to observe the sensitivity or specificity of the tool in relation to the full measure or a gold standard. Finally, the study was conducted in a closed refugee camp so the generalizability of the results may be limited.

### Implications

This is the first reported factor structure of the WHODAS 2.0 12-item Arabic version among Syrian refugees, and in a camp setting. Our findings are consistent with the original intention of the development of the WHODAS 2.0 12-item version, to be used as a screener; the internal reliability of the measures was acceptable, and a Cronbach's alpha above 0.7 indicated that it is additionally appropriate for use in a research setting. The exploratory and subsequent CFA provided evidence for a three-factor structure in this population. Further testing of the WHODAS 2.0 measure in refugee populations would be beneficial and would assist with the validation for further use of the tool.

## Data Availability

The data is initially available to the STRENGTHS Consortium to allow individual patient data meta-analyses of all PM+ trials conducted within this consortium. Following this, data will be freely available on request.

## References

[1] Miller KE, Rasmussen A. The mental health of civilians displaced by armed conflict: an ecological model of refugee distress. Epidemiol Psychiatr Sci 2017; 26: 129–38. 27040595 10.1017/S2045796016000172 PMC6998767

[2] Drescher A, Kiselev N, Akhtar A, Acarturk C, Bryant RA, Ilkkursun Z, Problems after flight: understanding and comparing Syrians’ perspectives in the Middle East and Europe. BMC Public Health 2021; 21: 1–12. 33849507 10.1186/s12889-021-10498-1 PMC8045311

[3] Li SS, Liddell BJ, Nickerson A. The relationship between post-migration stress and psychological disorders in refugees and asylum seekers. Curr Psychiatry Rep 2016; 18: 1–9. 27436307 10.1007/s11920-016-0723-0

[4] Schick M, Zumwald A, Knöpfli B, Nickerson A, Bryant RA, Schnyder U, Challenging future, challenging past: the relationship of social integration and psychological impairment in traumatized refugees. Eur J Psychotraumatol 2016; 7: 28057. 26886484 10.3402/ejpt.v7.28057 PMC4756625

[5] World Health Organization. International Classification of Functioning, Disability and Health: ICF. World Health Organization, 2001.

[6] Üstün TB, Chatterji S, Kostanjsek N, Rehm J, Kennedy C, Epping-Jordan J, Developing the World Health Organization Disability Assessment Schedule 2.0. Bull World Health Organ 2010; 88: 815–23. 21076562 10.2471/BLT.09.067231 PMC2971503

[7] Üstün TB, Kostanjsek N, Chatterji S, Rehm J. Measuring Health and Disability: Manual for WHO Disability Assessment Schedule WHODAS 2.0. World Health Organization, 2010.

[8] Federici S, Bracalenti M, Meloni F, Luciano JV. World Health Organization Disability Assessment Schedule 2.0: an international systematic review. Disabil Rehabil 2017; 39: 2347–80. 27820966 10.1080/09638288.2016.1223177

[9] Saltychev M, Katajapuu N, Bärlund E, Laimi K. Psychometric properties of 12-item self-administered World Health Organization Disability Assessment Schedule 2.0 (WHODAS 2.0) among general population and people with non-acute physical causes of disability - systematic review. Disabil Rehabil 2021; 43(6): 789–94. 31335215 10.1080/09638288.2019.1643416

[10] Abedzadeh-Kalahroudi M, Razi E, Sehat M, Asadi-Lari M. Psychometric properties of the World Health Organization Disability Assessment Schedule II -12 Item (WHODAS II) in trauma patients. Injury 2016; 47: 1104–8. 26710706 10.1016/j.injury.2015.11.046

[11] Andrews G, Kemp A, Sunderland M, Von Korff M, Ustun TB. Normative data for the 12 item WHO Disability Assessment Schedule 2.0. PLoS One 2009; 4: 6. 10.1371/journal.pone.0008343 PMC2791224 20020047

[12] Axelsson E, Lindsäter E, Ljótsson B, Andersson E, Hedman-Lagerlöf E. The 12-item self-report World Health Organization Disability Assessment Schedule (WHODAS) 2.0 administered via the internet to individuals with anxiety and stress disorders: a psychometric investigation based on data from two clinical trials. JMIR Ment Health 2017; 4: e58. 29222080 10.2196/mental.7497 PMC5741825

[13] Gaskin CJ, Lambert SD, Bowe SJ, Orellana L. Why sample selection matters in exploratory factor analysis: implications for the 12-item World Health Organization Disability Assessment Schedule 2.0. BMC Med Res Methodol 2017; 17: 40. 28283019 10.1186/s12874-017-0309-5 PMC5346210

[14] Papadopoulou M, Stasi S, Bakalidou D, Papageorgiou E, Tsokani A, Bratsi T, Psychometric properties of the 12-item World Health Organization Disability Assessment Schedule (WHODAS 2.0) in adult patients with motor disabilities. J Dev Phys Disabil, 2020; 32(5): 801–19.

[15] Saltychev M, Bärlund E, Mattie R, McCormick Z, Paltamaa J, Laimi K. A study of the psychometric properties of 12-item World Health Organization Disability Assessment Schedule 2.0 in a large population of people with chronic musculoskeletal pain. Clin Rehabil 2017; 31: 262–72. 26851249 10.1177/0269215516631385

[16] Schiavolin S, Ferroli P, Acerbi F, Brock S, Broggi M, Cusin A, Disability in Italian neurosurgical patients: validity of the 12-item World Health Organization Disability Assessment Schedule. Int J Rehabil Res 2014; 37: 267–70. 24802978 10.1097/MRR.0000000000000064

[17] Snell DL, Iverson GL, Panenka WJ, Silverberg ND. Preliminary validation of the World Health Organization Disability Assessment Schedule 2.0 for mild traumatic brain injury. J Neurotrauma 2017; 34: 3256–61. 28895491 10.1089/neu.2017.5234

[18] Sousa RM, Dewey ME, Acosta D, Jotheeswaran AT, Castro-Costa E, Ferri CP, Measuring disability across cultures the psychometric properties of the WHODAS II in older people from seven low- and middle-income countries. the 10/66 Dementia research group population-based survey. Int J Methods Psychiatr Res 2010; 19: 1–17. 10.1002/mpr.299 PMC2896722 20104493

[19] Subramaniam M, Abdin E, Vaingankar JA, Sagayadevan V, Shahwan S, Picco L, Validation of the World Health Organization Disability Assessment Schedule 2.0 among older adults in an Asian country. Singapore Med J 2020; 61: 246–53. 31197373 10.11622/smedj.2019049 PMC7905154

[20] Carlozzi NE, Kratz AL, Downing NR, Goodnight S, Miner JA, Migliore N, Validity of the 12-item World Health Organization Disability Assessment Schedule 2.0 (WHODAS 2.0) in individuals with Huntington disease (HD). Qual Life Res 2015; 24: 1963–71. 25636661 10.1007/s11136-015-0930-x PMC4497948

[21] Ćwirlej-Sozańska A, Sozański B, Kotarski H, Wilmowska-Pietruszyńska A, Wiśniowska-Szurlej A. Psychometric properties and validation of the polish version of the 12-item WHODAS 2.0. BMC Public Health 2020; 20: 1203. 32758211 10.1186/s12889-020-09305-0 PMC7409488

[22] Denu ZA, Yassin MO, Bisetegn TA, Biks GA, Gelaye KA. The 12 items Amharic version WHODAS-2 showed cultural adaptation and used to measure disability among road traffic trauma victims in Ethiopia. BMC Psychol 2021; 9: 1. 33388086 10.1186/s40359-020-00492-4 PMC7777354

[23] Hoehne A, Giguère CE, Herba CM, Labelle R. Évaluation du fonctionnement de patients présentant des troubles mentaux courants en milieu psychiatrique : Résultats au WHODAS-2. [Assessing functioning across common mental disorders in psychiatric emergency patients: results from the WHODAS-2.] Can J Psychiatry [Epub ahead of print] 23 Dec 2020. Available from: 10.1177/0706743720981200. PMC8689447 33353429

[24] Kimber M, Rehm J, Ferro MA. Measurement invariance of the WHODAS 2.0 in a population-based sample of youth. PLoS One 2015; 10: e0142385. 26565410 10.1371/journal.pone.0142385 PMC4643914

[25] Kirchberger I, Braitmayer K, Coenen M, Oberhauser C, Meisinger C. Feasibility and psychometric properties of the German 12-item WHO Disability Assessment Schedule (WHODAS 2.0) in a population-based sample of patients with myocardial infarction from the MONICA/KORA myocardial infarction registry. Popul Health Metr 2014; 12(1): 1–13.24479861

[26] MacLeod MA, Tremblay PF, Graham K, Bernards S, Rehm J, Wells S. Psychometric properties and a latent class analysis of the 12-item World Health Organization Disability Assessment Schedule 2.0 (WHODAS 2.0) in a pooled dataset of community samples. Int J Methods Psychiatr Res 2016; 25: 243–54. 27634553 10.1002/mpr.1523 PMC6860311

[27] Marx BP, Wolf EJ, Cornette MM, Schnurr PP, Rosen MI, Friedman MJ, Using the WHODAS 2.0 to assess functioning among veterans seeking compensation for posttraumatic stress sisorder. Psychiatr Serv 2015; 66: 1312–7. 26278226 10.1176/appi.ps.201400400

[28] Park SH, Demetriou EA, Pepper KL, Song YJC, Thomas EE, Hickie IB, Validation of the 36-item and 12-item self-report World Health Organization Disability Assessment Schedule II (WHODAS-II) in individuals with autism spectrum disorder. Autism Res 2019; 12: 1101–11. 31033250 10.1002/aur.2115

[29] Saltychev M, Mattie R, McCormick Z, Laimi K. Confirmatory factor analysis of 12-Item World Health Organization Disability Assessment Schedule in patients with musculoskeletal pain conditions. Clin Rehabil 2017; 31: 702–9. 27260763 10.1177/0269215516652930

[30] Silveira C, Souza RT, Costa ML, Parpinelli MA, Pacagnella RC, Ferreira EC, Validation of the WHO Disability Assessment Schedule (WHODAS 2.0) 12-item tool against the 36-item version for measuring functioning and disability associated with pregnancy and history of severe maternal morbidity. Int J Gynaecol Obstet 2018; 141 (Suppl 1): 39–47. 29851113 10.1002/ijgo.12465 PMC6001571

[31] Tompke BK, Tang J, Oltean II, Buchan MC, Reaume SV, Ferro MA. Measurement invariance of the WHODAS 2.0 across youth with and without physical or mental conditions. Assessment 2020; 27: 1490–501. 30501405 10.1177/1073191118816435

[32] Younus MI, Wang DM, Yu FF, Fang H, Guo X. Reliability and validity of the 12-item WHODAS 2.0 in patients with Kashin-Beck disease. Rheumatol Int 2017; 37: 1567–73. 28439626 10.1007/s00296-017-3723-4

[33] Tol WA, Barbui C, Galappatti A, Silove D, Betancourt TS, Souza R, Mental health and psychosocial support in humanitarian settings: linking practice and research. Lancet 2011; 378: 1581–91. 22008428 10.1016/S0140-6736(11)61094-5 PMC3985411

[34] Tay AK, Rees S, Miah MAA, Khan S, Badrudduza M, Morgan K, Functional impairment as a proxy measure indicating high rates of trauma exposure, post-migration living difficulties, common mental disorders, and poor health amongst Rohingya refugees in Malaysia. Transl Psychiatry 2019; 9: 213. 31477686 10.1038/s41398-019-0537-z PMC6718407

[35] Rasmussen A, Nguyen L, Wilkinson J, Vundla S, Raghavan S, Miller KE, Rates and impact of trauma and current stressors among Darfuri refugees in Eastern Chad. Am J Orthopsychiatry 2010; 80: 227–36. 20553516 10.1111/j.1939-0025.2010.01026.x PMC2920620

[36] Al-Rousan T, Schwabkey Z, Jirmanus L, Nelson BD. Health needs and priorities of Syrian refugees in camps and urban settings in Jordan: perspectives of refugees and health care providers. East Mediterr Health J 2018; 24: 243–53. 29908019 10.26719/2018.24.3.243

[37] UNHCR. Syria Regional Refugee Response. UNHCR, 2020 (https://data2.unhcr.org/en/situations/syria/location/36).

[38] UNHCR. Jordan: Azraq Refugee Camp. UNHCR, 2020.

[39] Akhtar A, Giardinelli L, Bawaneh A, Awwad M, Naser H, Whitney C, Group problem management plus (gPM+) in the treatment of common mental disorders in Syrian refugees in a Jordanian camp: study protocol for a randomized controlled trial. BMC Public Health 2020; 20: 1–8. 32216762 10.1186/s12889-020-08463-5 PMC7098148

[40] Bontempo R. Translation fidelity of psychological scales: an item response theory analysis of an individualism-collectivism scale. J Cross Cult Psychol 1993; 24: 149–66.

[41] Kessler RC, Andrews G, Colpe LJ, Hiripi E, Mroczek DK, Normand SLT, Short screening scales to monitor population prevalences and trends in non-specific psychological distress. Psychol Med 2002; 32: 959–76. 12214795 10.1017/s0033291702006074

[42] Easton SD, Safadi NS, Wang Y, Hasson RG. 3rd. The Kessler Psychological Distress Scale: translation and validation of an Arabic version. Health Qual Life Outcomes 2017; 15: 215. 29078774 10.1186/s12955-017-0783-9 PMC5658946

[43] Sulaiman-Hill CMR, Thompson SC. Selecting instruments for assessing psychological wellbeing in Afghan and Kurdish refugee groups. BMC Res Notes 2010; 3: 237. 20825681 10.1186/1756-0500-3-237 PMC2949661

[44] Nunnally JC. Psychometric Theory (2nd edn). McGraw-Hill, 1978.

[45] Osborne J, Costello A, Kellow J. Best Practices in Quantitative Methods. SAGE Publications, Inc, 2008.

[46] Bentler PM. Comparative fit indexes in structural models. Psychol Bull 1990; 107: 238. 2320703 10.1037/0033-2909.107.2.238

[47] IBM. IBM SPSS Statistics for Windows, Version 26.0. IBM Corp, 2019.

[48] Bollen KA. Structural Equations with Latent Variables. John Wiley & Sons, 1989.

[49] Comrey AL. Common methodological problems in factor analytic studies. J Consult Clin Psychol 1978; 46: 648.

